# Robust Machine Learning-Based Correction on Automatic Segmentation of the Cerebellum and Brainstem

**DOI:** 10.1371/journal.pone.0156123

**Published:** 2016-05-23

**Authors:** Jun Yi Wang, Michael M. Ngo, David Hessl, Randi J. Hagerman, Susan M. Rivera

**Affiliations:** 1 Center for Mind and Brain, University of California-Davis, Davis, California, United States of America; 2 Medical Investigation of Neurodevelopmental Disorders (MIND) Institute, University of California-Davis Medical Center, Sacramento, California, United States of America; 3 Department of Psychiatry and Behavioral Sciences, University of California-Davis, School of Medicine, Sacramento, California, United States of America; 4 Department of Pediatrics, University of California-Davis, School of Medicine, Sacramento, California, United States of America; 5 Department of Psychology, University of California-Davis, Davis, California, United States of America; University of Pécs Medical School, HUNGARY

## Abstract

Automated segmentation is a useful method for studying large brain structures such as the cerebellum and brainstem. However, automated segmentation may lead to inaccuracy and/or undesirable boundary. The goal of the present study was to investigate whether SegAdapter, a machine learning-based method, is useful for automatically correcting large segmentation errors and disagreement in anatomical definition. We further assessed the robustness of the method in handling size of training set, differences in head coil usage, and amount of brain atrophy. High resolution T1-weighted images were acquired from 30 healthy controls scanned with either an 8-channel or 32-channel head coil. Ten patients, who suffered from brain atrophy because of fragile X-associated tremor/ataxia syndrome, were scanned using the 32-channel head coil. The initial segmentations of the cerebellum and brainstem were generated automatically using Freesurfer. Subsequently, Freesurfer’s segmentations were both manually corrected to serve as the gold standard and automatically corrected by SegAdapter. Using only 5 scans in the training set, spatial overlap with manual segmentation in Dice coefficient improved significantly from 0.956 (for Freesurfer segmentation) to 0.978 (for SegAdapter-corrected segmentation) for the cerebellum and from 0.821 to 0.954 for the brainstem. Reducing the training set size to 2 scans only decreased the Dice coefficient ≤0.002 for the cerebellum and ≤ 0.005 for the brainstem compared to the use of training set size of 5 scans in corrective learning. The method was also robust in handling differences between the training set and the test set in head coil usage and the amount of brain atrophy, which reduced spatial overlap only by <0.01. These results suggest that the combination of automated segmentation and corrective learning provides a valuable method for accurate and efficient segmentation of the cerebellum and brainstem, particularly in large-scale neuroimaging studies, and potentially for segmenting other neural regions as well.

## Introduction

The human brain contains three major components that are heavily interconnected: the cerebrum, cerebellum and brainstem. The cerebrum has long been considered as the primary site for cognition while the cerebellum has been implicated in movement coordination; however, the recent paradigm shift for recognizing the importance of large-scale distributed brain networks for brain function and dysfunction [1] ignites interest in investigating the role of the cerebellum in cognition [2]. The involvement of the cerebellum in cognitive and emotional processing is supported by the identical cytoarchitecture across cerebellar cortex and reciprocal and functionally segregated connections with almost all areas of the neocortex. These two unique anatomical features of the cerebellum suggest its uniformity and modularity in information processing for various brain functions [3–5]. Additional support for this claim comes from phylogeny, functional neuroimaging, and lesion studies. Specifically, phylogenetic studies showed the expansion of the cerebellum with the frontal lobe in primates [2,6]. Functional neuroimaging studies revealed the co-activation of the cerebellum with the prefrontal and parietal lobes independent of motor activity in a variety of cognitive tasks [7–9]. Finally, lesion studies displayed the range of cognitive and sensorimotor problems experienced by patients of cerebellar cognitive affective syndrome due to cerebellar lesions [10,11]. In spite of the general agreement of the cerebellum’s involvement in cognition, more work needs to be done to unravel the mechanisms underlying this involvement [2].

The brainstem connects the cerebrum and cerebellum and carries information between these two structures and the spinal cord. In addition to functioning as a conduit, the brainstem contains various nuclei that serve cranial nerve functions and integrative functions for vital physiological processes including respiration, heartbeat, circulation, arousal and movement [12,13]. Some brainstem nuclei are also sources of monoamine neurotransmitters (norepinephrine, dopamine, and serotonin). These nuclei form extensive closed-loop circuits with various cortical areas, regulating and even initiating movement and cognitive processing [12,14]. Dysregulated neuromodulatory systems have been implicated in major psychiatric disorders such as schizophrenia, depression and anxiety disorders and neurodegenerative disorders such as Parkinson’s disease and Alzheimer’s disease [12,14]. Consistent with its wide cortical and subcortical projections and range of functions that it serves, brainstem injury often results in motor deficit and long-lasting cognitive impairment [15–17].

In light of recent recognition of the involvement of the cerebellum and brainstem in a diverse set of functions, studying these structures is critical for our understanding of neural substrates for motor, cognitive and affective processing and the effect of changes in these two structures from development, aging, and various psychiatric and neurological disorders [18–20]. Brain structure segmentation is an important method in neuroimaging analyses, which not only produces volumetric data for analyzing structural changes in various conditions, but also allows for visualization of the anatomic structures and subsequent functional or structural neuroimaging analyses, e.g. diffusion tensor imaging. Segmentations of the cerebellum and brainstem can be performed manually, automatically by a computer program, or semi-automatically combining both manual and automatic procedures [21].

Manual tracing has often served as the gold standard because this method can produce highly accurate results depending on the rater’s expertise. It is flexible and can accommodate variations in anatomical definitions; however, manual tracing is time consuming and challenging especially for a big structure, such as the cerebellum with thin folia, or for processing a large dataset [22,23]. Manual tracing also requires extensive rater training and may result in inconsistency because of intra- and inter-rater variability.

To improve efficiency, accuracy and consistency, various automated methods have been developed for segmenting the cerebellum and brainstem [21,24]. Some of these methods are free and publicly available, including probabilistic atlas-based FreeSurfer [25], ANTs/Atropos [26], and SPM/SUIT [27], multi-atlas based MAGeT [28], multi-atlas with joint label fusion [29] and surfaced-based shape and appearance modelling—FSL/FIRST [30]. Two of the methods [27,28] are specifically created for segmenting the cerebellum in lobules. Automated segmentation has the advantage of producing consistent results with little human intervention and is particularly desirable for segmenting multiple structures or dealing with large datasets. Automatic segmentation heavily relies on one or more the following features: location, shape, and MRI signal intensity of the structure and the surrounding areas. It often requires the propagation of segmentation labels from one or more template images to a new set of images [21,24]. The drawback of this method is the lack of flexibility for accommodating differences in anatomical definitions or in features for segmentation due to pathological conditions [21,24].

Recently, a machine-learning based method, SegAdapter, has been created for improving the accuracy and flexibility of automated segmentation by correcting consistent errors that frequently occur during automated segmentation [31,32]. This post-processing segmentation method implements AdaBoost, a machine-learning algorithm [33], to learn spatial, intensity and contextual patterns of segmentation errors in automated segmentation and applies the learning to correct errors in new images effectively. The goal of the current study was to apply the corrective learning on cerebellum and brainstem segmentations generated by FreeSurfer. FreeSurfer is a popular neuroimaging tool for automated atlas-based segmentation of subcortical structures [25] as well as cortical surface reconstruction, parcellation (also atlas-based), and inflation [34–37]. However, its segmentation of the brainstem is incomplete, omitting important structures including the substantia nigra in the midbrain. The segmentation also contains other errors that have been previously reported [38,39]. We tested whether SegAdapter was useful for correcting segmentation errors from FreeSurfer and whether the results were affected by size of the training set, differences between the training set and the testing set, such as head coil used during scan acquisition, and amount of brain atrophy. We found significant improvement in segmentation of both the cerebellum and brainstem and robustness of the corrective learning across different conditions.

This study is a part of two ongoing research projects on the fragile X premutation. The fragile X mental retardation 1 (*FMR1*) gene encodes fragile X mental retardation protein (FMRP), which is important for synaptic development and plasticity [40]. The gene contains a CGG repeat element in its non-coding region, with a dynamic repeat size affecting gene and protein expressions and causing different types of brain disorders. Normal alleles typically contain 5–44 CGG repeats. While CGG expansion to the full mutation range (>200 repeats) leads to the developmental disorder, fragile X syndrome [41], CGG expansion to the premutation range (55–200 repeats) causes a late-onset neurodegenerative disorder, Fragile X-Associated Tremor/Ataxia Syndrome (FXTAS), which typically affects older (>50 years) premutation carriers [42,43]. The principal features of FXTAS include intention tremor, cerebellar ataxia, Parkinsonism, autonomic dysfunction and cognitive impairment. Both neuropathological and neuroimaging studies have revealed severe damage of the cerebellum and brainstem in FXTAS [44–50]. Thus, the inclusion of patients with FXTAS allowed us to examine the effectiveness of our proposed method in a condition which causes cerebellum and brainstem atrophy.

## Materials and Methods

### Research participants and imaging

The two research projects associated with this study have been approved by the Institutional Review Board (IRB) at University of California Davis. Written informed consent has been acquired from all research participants according to the consent procedures approved by the IRB at University of California Davis. High-resolution T1-weighted images were acquired on a Siemens Trio 3T MRI scanner (Siemens Medical Solutions, Erlangen, Germany). We randomly selected 40 male participants of which 10 younger (age 18–42 years) healthy participants carrying normal *FMR1* alleles were scanned using an 8-channel head coil while the remaining 30 participants were scanned using a 32-channel head coil. These 30 participants were 10 younger (age 21–42 years) and 10 older healthy controls (age 56–82 years) carrying normal *FMR1* alleles, and 10 older *FMR1* premutation carriers (age 64–77 years) who have been diagnosed with FXTAS. See Table 1 for participants’ demographic and scanning information. High-resolution T1-weighted 3D magnetization prepared rapid gradient echo (MPRAGE) images were obtained in 192 sagittal slices of 1 mm thickness (no gap) with FOV 256 mm, 256 × 256 matrix, TR of 2,170 ms, TE of 4.82 ms, and 7° flip angle. The imaging protocols were the same for scans acquired with 8- and 32-channel head coil, although the scanner went through a major hardware and software upgrade in late 2009 followed by the switch of the head coil from 8-channel to 32 channel in early 2010. All 10 younger participants who were scanned using the 8-channel head coil were scanned before the scanner upgrade.

**Table 1:** The participants’ demographic and MRI scan acquisition information.

*N*	Age (Years)	Age Range (Years)	*FMR1* Allele	Head Coil	Scan Year
10	29.1 ± 8.7	18–42	Normal	8 channel	2008–2009
10	31.1 ± 8.2	21–42	Normal	32 channel	2010–2012
10	70.3 ± 7.3	56–82	Normal	32 channel	2010–2013
10	67.9 ± 5.6	64–77	FX Premutation	32 channel	2010–2013

### Segmentation generation

The raw dicom files of the MPRAGE images were inspected and transformed to axial slices in ANALYZE format using DTI Studio (http://cmrm.med.jhmi.edu/) and then processed in FreeSurfer (http://freesurfer.net/), which contains a set of software tools for performing structural and functional neuroimaging analyses. To conduct the cerebellum and brainstem segmentations, the complete surface-based process was run automatically, using the default parameters. The steps included affine Talairach registration, B1 bias field estimation followed by normalization of MRI signal intensity, skull-stripping using a deformable template model, gray and white matter segmentation based on intensity and neighbor constraints, generation of the white matter and gray matter surfaces, and cortical and subcortical labeling using probabilistic atlas-based segmentation [34–37]. After the surface-based process was completed, we inspected the results to correct major morphological errors. One common error we encountered was the exclusion of brain tissues or inclusion of non-brain tissues during the skull strip step. We fixed the error by either modifying the brain mask or adjusting the watershed parameters for skull stripping, followed by regeneration of the white matter and gray matter surfaces and labels.

The FreeSurfer segmentations of the cerebellum and brainstem was manually corrected for errors and modified according to classic anatomic definitions [12], recently published methods [23,38], and online resources showing segmentations in sagittal view (Laboratory of Neuro Imaging, LONI, University of Southern California, USA, http://resource.loni.usc.edu/resources/downloads/research-protocols/masking-regions/brainstem/) and all three views (IMAIOS SAS, France, https://www.imaios.com/en/e-Anatomy/Head-and-Neck/Brain-MRI-3D). The outlines of the two structures were modified in all three views to maintain smoothness. The modification was performed on aseg.mgz maps using tkmedit, a software tool provided by FreeSurfer for inspecting and editing images in FreeSurfer internal format, mgz.

To reduce the amount of training, we employed two groups of raters, the junior and expert raters, for segmentation correction. The junior raters corrected obvious errors that required minimum training including dura and surrounding tissues mislabeled as cerebellum, missing cerebellar and brainstem voxels, and brainstem voxels mislabeled as cerebellum in the midbrain and medullar oblongata where clear boundaries exist. The expert raters completed all required corrections, including the interface between the cerebellum and brainstem that showed similar signal intensity as well as the top portion of the brainstem that needed to be extended to include the substantia nigra. The strategy was to combine shape knowledge with subtle contrast in signal intensities in some of the slices to estimate the interface. This was performed on selective slices from all 3 views. The result was partially relabeled cerebellum and brainstem on slices where the complete interface could be determined by interpolation. After relabeling on all slices, the interface was further corrected in all three views to maintain the shape and smoothness for both structures. To maintain consistency amongst the expert raters, the final segmentations in all scans was checked by a single expert rater, J.Y.W.

### Segmentation correction

We supplied three sets of images to the corrective learning tool, SegAdapter (http://www.nitrc.org/projects/segadapter/): (1) segmentation from the automated FreeSurfer process, (2) manually edited segmentation and (3) MPRAGE scans, with signal intensity squared to improve contrast. To generate the segmentation masks, the labels of the cerebellum and brainstem in the initial and edited aseg.mgz files were resampled into the original MPRAGE space using the command, mri_label2vol. Next, the cerebellar hemisphere and white matter labels were merged due to substantial errors observed in segmenting these two types of tissues in many scans. The merge was performed using the command, fslmaths, from FSL (http://fsl.fmrib.ox.ac.uk/fsl/fslwiki/), another well-known, publicly available tool set for analyzing functional and structural images. The left and right cerebellar labels were also merged using the same command, fslmaths, followed by combining the cerebellum and brainstem labels (with different values) into a single mask for corrective learning. The motion-corrected and intensity-normalized T1.mgz scans from FreeSurfer were supplied as feature files for providing patterns of signal intensities of the labels. These scans were transformed into the original MPRAGE space using mri_convert and signal intensity squared using fslmaths.

Separate corrective learning in SegAdapter needed to be performed for each of the labels and background using three parameters: dilation radius for each label’s working ROI; feature patch size applied to the MPRAGE images; and sampling rate—the rate of voxels in the working ROI to be included for learning [32]. The working ROI was generated by 3D voxel dilation from the Freesurfer-generated segmentation which should cover majority of the voxels assigned labels by manual segmentation. Voxels in the working ROI were evaluated and mislabeled voxels were identified as targets for corrective learning. For each label and the background, corrective learning was then performed to distinguish between the following two classes of mislabeled voxels: (1) voxels assigned the corresponding label or background; and (2) voxels assigned labels other than the corresponding label or background by the manual segmentation. For each target voxel in the working ROI, a feature patch was generated by voxel dilation according to user-supplied feature patch size. The corrective learning was then performed by utilizing the following three features of all voxels in the feature patch: signal intensity, segmentation label generated by the automated segmentation, and coordinates. The best features for classification were selected and combined into a single strong classifier for each label. Since the corrective learning is a memory intensive computing process affected by all of the three parameters (i.e. dilution radius, feature patch size, and sampling rate) plus the size of the training set, we tested various combinations of the parameters and selected feasible values before the actual learning. The number of iterations was set to 500. During testing, the three classifiers, corresponding to the two labels and background, assigned new labels to voxels in the working ROI, and the labels with the strongest responses were selected as the final labels [31,32].

To evaluate the performance of the corrective learning, we performed 10 cross-validation experiments. We randomly selected (2 or 5) scans to be included in the training set and the remaining scans (8 or 5) became the testing set. We applied the corrective learning to scans in the testing set by supplying the automated segmentation and the MPRAGE scans with squared signal intensity. Visualization and 3D reconstruction of the cerebellum and brainstem segmentation were performed in online free software, ITK-SNAP (http://www.itksnap.org/) [51]. The volumes of segmented cerebellum and brainstem, and the volume overlap between SegAdpater corrected and manually corrected segmentation were calculated using fslstats command from FSL. Voxel-wise spatial Dice overlap was calculated as *2|A∩B| / (|A|+|B|)*, which measures similarity between two segmentations with value ranging from 0 (no spatial overlap between two segmentations) to 1 (100% overlap). The corrective learning was conducted on an Intel(R) Xeon(R) E5-2640 v2 computer with 32 processing units at CPU of 2.0 GHz and memory of 132 GB.

### Statistical analysis

We performed paired *t*-tests for comparing the effectiveness of corrective learning in different conditions using the open-source statistical package R (http://www.r-project.org/). For testing intra-rater reliability, we randomly selected 8 scans and manually corrected FreeSurfer segmentation errors twice. Intra-class correlation coefficient (ICC) for absolute agreement in volumes was performed using the “psych” package for R (http://cran.r-project.org/web/packages/psych/).

## Results

### Segmentation

**Fig 1:**
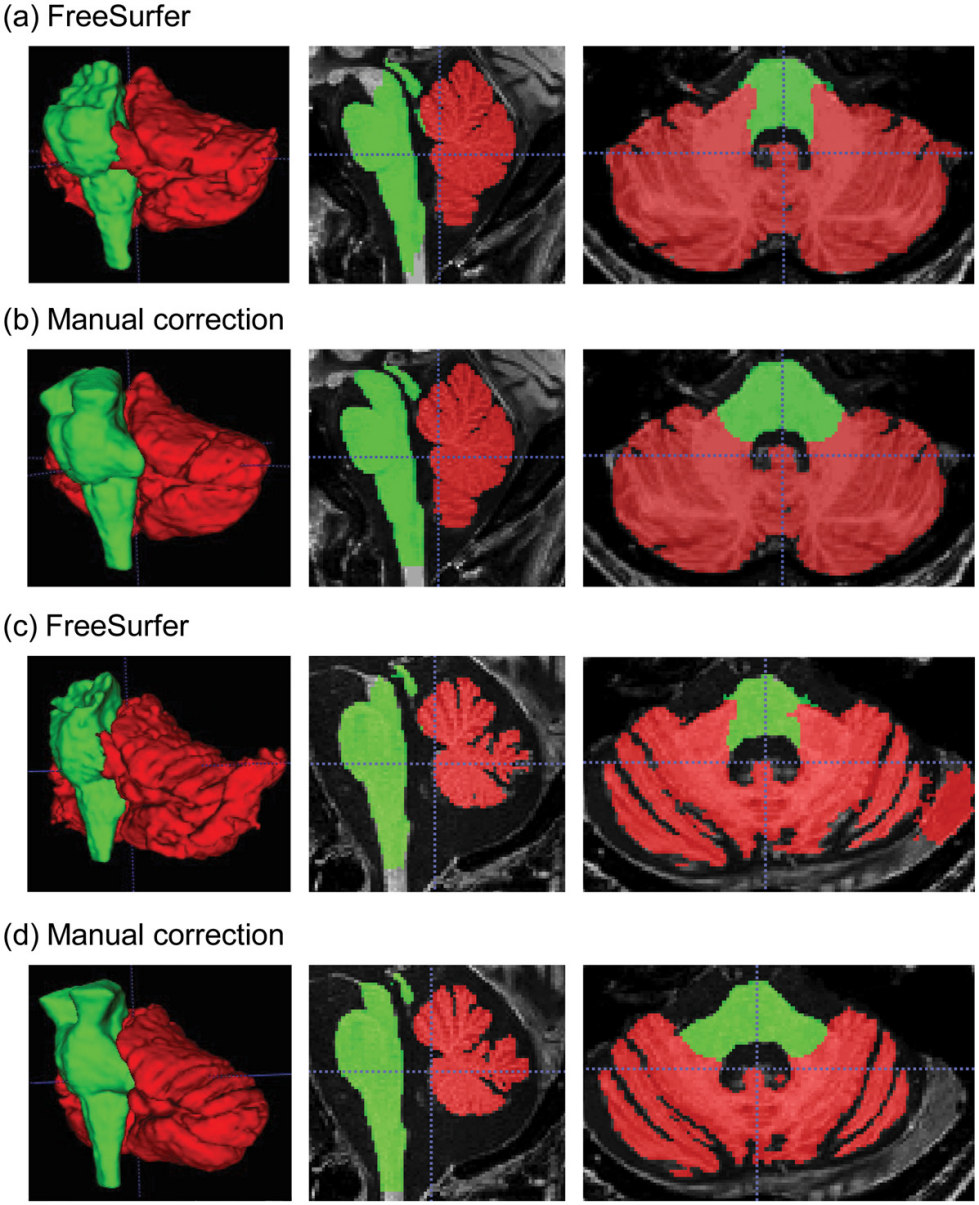
A sample of segmented cerebellum and brainstem. The left column shows the 3D-reconstructed structures while the middle column show a sagittal view and the right column shows an axial view. (a) The original segmentation from FreeSurfer automated process for a healthy control; (b) the corresponding manually corrected segmentation; (c) the FreeSurfer segmentation for a patient with neurodegeneration; and (d) the corresponding manually correction segmentation. Note the top of the brainstem is filled in after manual correction as well as the correction of the cerebellar-brainstem interface and removal of non-brain tissue from cerebellar labelling. Red, cerebellum; lime, brainstem.

Manual correction of segmentation error in the cerebellum and brainstem took about 2–4 hours to complete for each brain. Fig 1 shows samples of the original segmentation from FreeSurfer (Fig 1a and 1c) and manually corrected segmentation (Fig 1b and 1d) for a young healthy control (Fig 1a and 1b) and a FXTAS patient with brain atrophy (Fig 1c and 1d). Intra-rater reliability for error correction measured using ICC (for absolute agreement) was excellent, 1.00 (95% confidence interval, CI, [0.93,1.00]) for cerebellar volume and 0.99 (95% CI [0.99, 1.00]) for brainstem volume. The Dice coefficients were 0.984 ± 0.003 for cerebellum and 0.977 ± 0.003 for brainstem.

**Fig 2:**
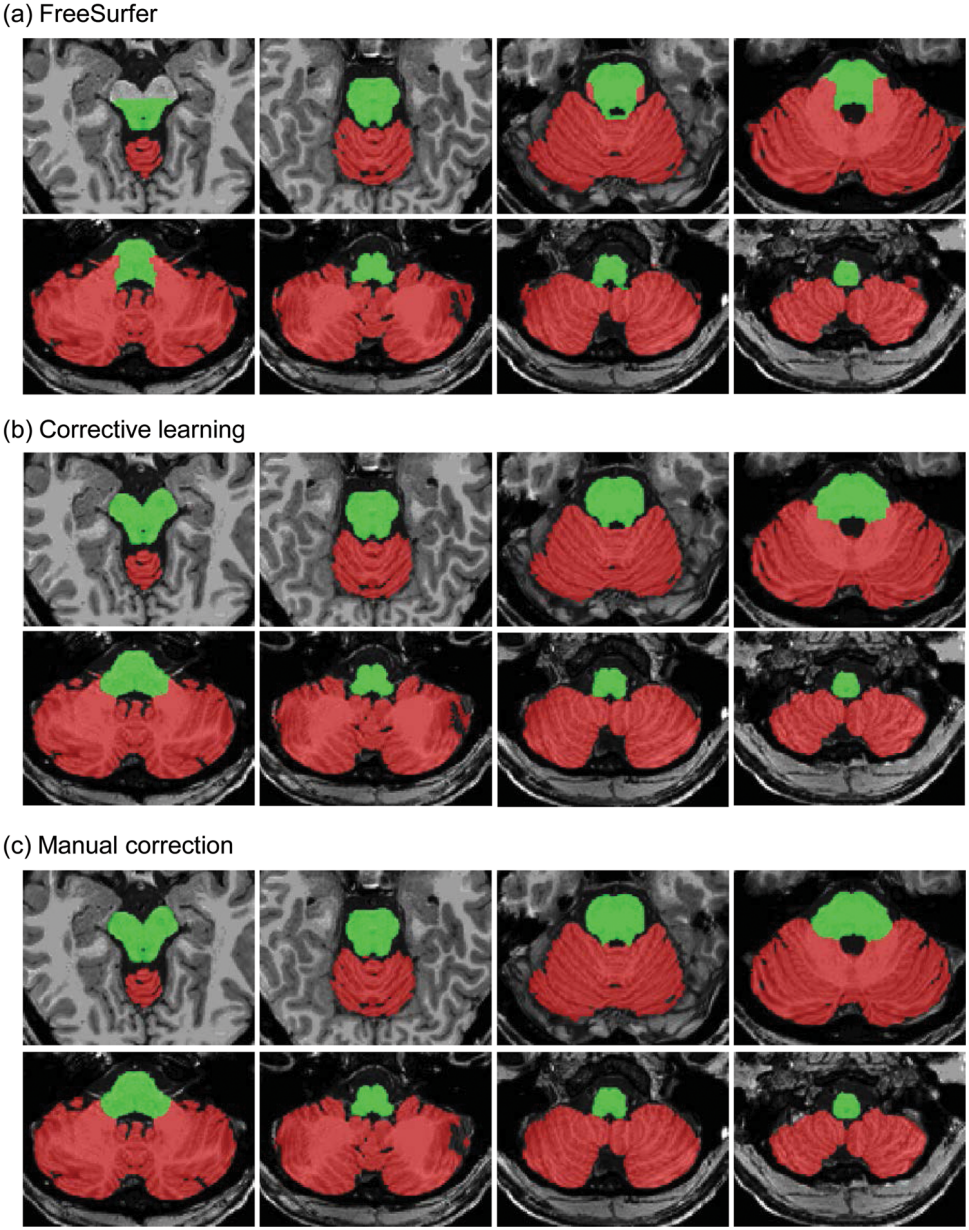
Cerebellum and brainstem segmentation in an axial view. (a) Segmentation from FreeSurfer automated process; (b) segmentation after corrective learning on a test scan using a training set of 5 scans; (c) manually edited segmentation. Red, cerebellum; lime, brainstem.

For corrective learning, the values of four parameters need to be chosen: dilation radius of the working ROI, feature patch size for MPRAGE, sampling rate and training set size. We found that the optimal dilation radius was 6 voxels for both the cerebellum and brainstem. Increase in the dilation radius beyond 6 voxels created a ring around the brainstem. Consequently, we set the feature patch size to 6×6×6 voxels. Due to the memory constraints during corrective learning, only 2 scans were allowed in the training set at 10% sampling rate for the cerebellum and background labels. At 5% sampling rate, corrective learning was conducted successfully with 5 scans in the training set. Consequently, we compared corrective learning between 2 and 5 scans in the training set at 10% and 5% sampling rate, respectively, for the cerebellum and background labels, and at 20% and 10%, respectively, for the brainstem label. In both settings, the training took about 8–10 hours for one set of scans while learning took only a few minutes for each scan. Fig 2 displays the axial slices of the segmentation from FreeSurfer (Fig 2a), corrective learning using a 5-scan training set on one of the scans from the test set (Fig 2b), and manual correction (Fig 2c). The improvement in corrective learning generated segmentation over FreeSurfer segmentation was visually observable for both structures. The residual consistent errors after corrective learning were missing voxels at the top of the brainstem and brainstem voxels mislabeled as cerebellum at the interface between the cerebellum and brainstem in the pons.

### Corrective learning using training set of 2 and 5 scans

**Fig 3:**
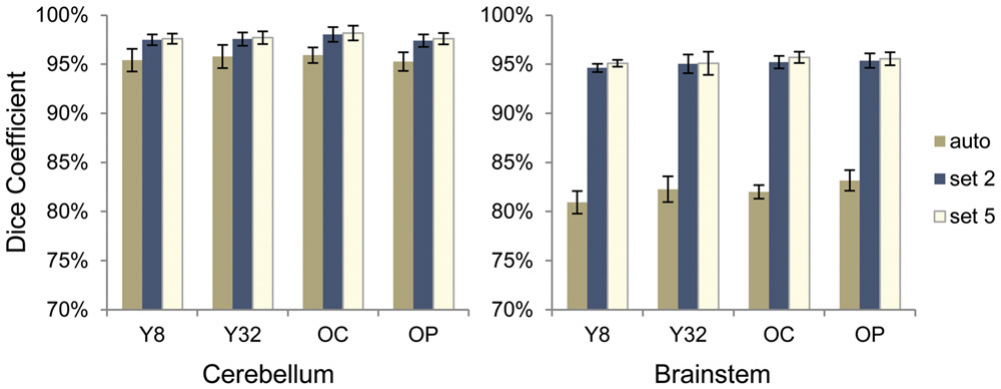
Dice coefficient for cerebellar and brainstem segmentations of the test scans against manual correction. The data shown in the graphs are from FreeSurfer automated process (auto), corrective learning using a training set of 2 scans (set 2) and corrective learning using a training set of 5 scans (set 5). There are four groups of participants: Y8, the younger healthy control group scanned using an 8 channel head coil; Y32, the younger healthy control group scanned using a 32 channel head coil; OC, the older healthy control group scanned using a 32 channel head coil; and OP, the older patient group with neurodegeneration scanned using a 32 channel head coil. Error bars indicate ±1 standard deviation.

Fig 3 shows the Dice coefficients of FreeSurfer automated process and segmentation after corrective learning of the test sets using the manual correction as the gold standard. For the 4 experimental groups, the Dice coefficients of FreeSurfer segmentation with manual correction were 0.952–0.959 (SD 0.008–0.012) for the cerebellum and 0.809–0.832 (SD 0.007–0.013) for the brainstem. After corrective learning, Dice coefficient of the test sets was significantly improved in all four groups (*t* = 8.8–69, *df* = 9, *p*-value <0.001), increased to 0.976–0.982 (SD 0.005–0.008) for the cerebellum and 0.951–0.957 (SD 0.003–0.012) for the brainstem when using the training set of 5 scans (Fig 3). Using the training set of 2 scans showed a small decline in Dice overlap in the test set from the use of the training set of 5 scans, which were 0.974–0.980 (SD 0.005–0.007) for cerebellum and 0.946–0.954 (SD 0.004–0.009) for brainstem.

We also compared the accuracy of volume measurements from FreeSurfer segmentation and SegAdapter corrected segmentation in the test set using the manually corrected segmentation as the gold standard. While the FreeSurfer segmentation showed ~5% higher cerebellar volume and ~25% lower brainstem volume compared to manually corrected volumes for the four groups, the volume differences were reduced to ~1% higher for cerebellum and ~3% lower for brainstem after the corrective learning (Table 2). Consistently, paired *t*-tests revealed no statistical differences in volumes between corrective learning and manual correction in the cerebellum (*t* = 1.1–1.7, *df* = 9, *ns*); however, significant differences were found in the brainstem (*t* = 3.2–4.7, *df* = 9, *p* = 0.001–0.010). Combining the 3 groups, ICC (for absolute agreement) was 0.99 (95% CI [0.98, 1.00]) between cerebellar volumes obtained from manual editing and corrective learning, and was 0.95 (95% CI [0.52, 0.98]) between brainstem volumes obtained using these two methods. In contrast, ICC was 0.94 (95% CI [0.045, 0.99]) for cerebellar volumes and 0.31 (95% CI [-0.015, 0.70]) for brainstem volumes between Freesurfer and manual segmentations.

**Table 2:** Cerebellum and brainstem volume (ml) calculated after FreeSurfer automated process, manual correction and corrective learning in the test set using training set of 2 and 5 scans.

	FreeSurfer	Manual Correction	Training Set of 2 Scans	Training Set of 5 Scans
Cerebellum				
Younger controls, 8 channel	140 ± 13*	132 ± 12	133 ± 12	133 ± 12
Younger controls, 32 channel	150 ± 17*	144 ± 16	145 ± 17	145 ± 17
Older controls, 32 channel	130 ± 12*	124 ± 10	125 ± 10	125 ± 10
Older patients, 32 channel	115 ± 16*	110 ± 15	112 ± 15	111 ± 15
Brainstem				
Younger controls, 8 channel	22.8 ± 2.0*	31.1 ± 2.6	30.1 ± 2.3*	30.2 ± 2.3*
Younger controls, 32 channel	26.1 ± 4.0*	34.5 ± 5.3	33.2 ± 4.4*	33.2 ± 4.4*
Older controls, 32 channel	23.5 ± 1.9*	31.7 ± 2.4	30.6 ± 2.2*	30.8 ± 2.3*
Older patients, 32 channel	20.7 ± 2.6*	27.2 ± 3.6	26.4 ± 3.2*	26.4 ± 3.2*

*, significant at FDR 5% compared with manual correction, *p* ≤ 0.010.

### The effect of head coil, aging, and neurodegenerative process

**Fig 4:**
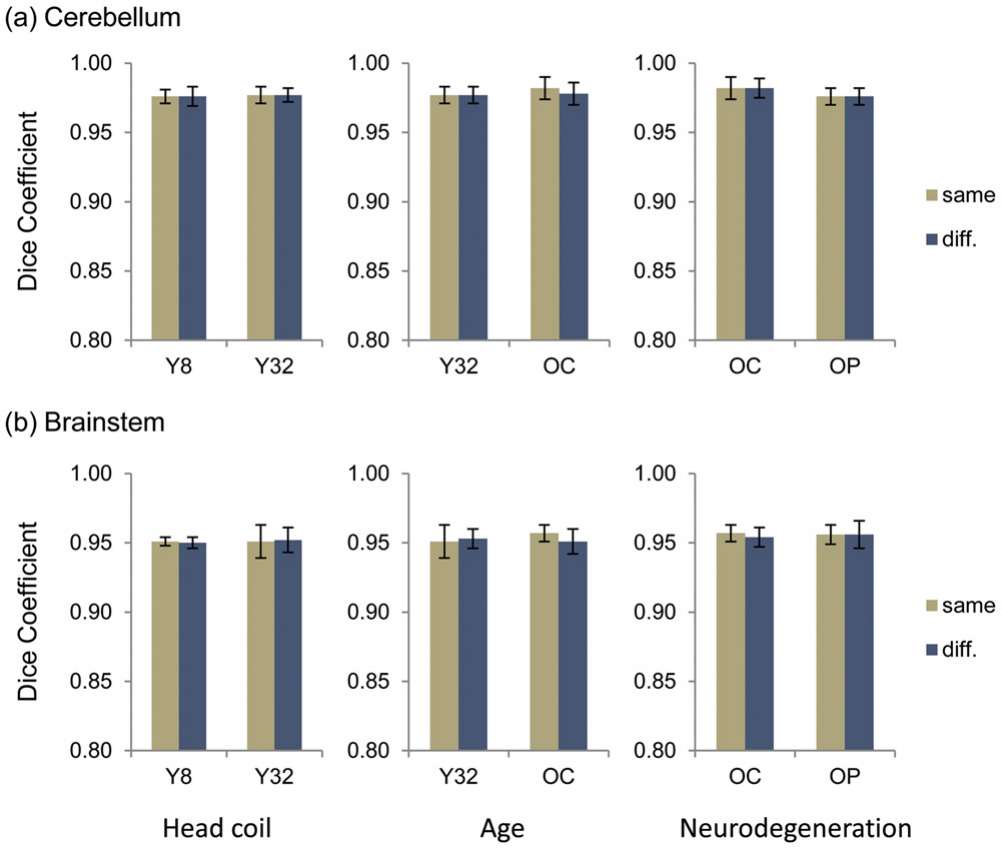
Dice coefficient for testing the effect of head coil used during scan acquisition and brain atrophy due to aging or neurodegenerative process on segmentation correction on (a) cerebellar segmentation and (b) brainstem segmentation. The columns of paired bar graphs, from left to right, show the effect on Dice coefficient due head coil differences, aging, and neurodegenerative process, respectively. **Y8** refers to the younger healthy control group scanned using an 8 channel head coil. **Y32** refers to the younger healthy control group scanned using a 32 channel head coil. **OC** refers to the older healthy control group scanned using a 32 channel head coil. **OP** refers to the older patient group with neurodegeneration scanned using a 32 channel head coil. Error bars indicate ±1 standard deviation. “Same” indicates that the training set and the testing set contained scans from the same group; and “diff.” indicated that the training set contained scans from a comparison group of the testing set.

We further examined the effect of head coil used during image acquisition and brain atrophy caused by aging or the involvement of neurodegenerative process on corrective learning. We applied corrective learning to scans from a different group. For instance, to test the effect of head coil, we applied corrective learning generated from the younger 8-channel head coil group to the scans of the younger 32-channel group and *vice versa*. Fig 4 exhibits the group average and standard deviation of Dice coefficients in each condition. All conditions had trivial effect on corrective learning, which reduced <0.01 Dice coefficient between corrective learning and manual correction. We subsequently performed paired *t*-test to assess statistical significance of the reduction in Dice coefficient. The results indicated sensitivity of the older control group to the applications of corrective learning from both the younger control group and the FXTAS patient group. Only the older control group showed significant effect of age for cerebellar segmentation (*t* = 8.5, *df* = 9, *p* < 0.001) as well as significant effect of age (*t* = 2.95, *df* = 9, *p* = 0.016) and amount of brain atrophy (*t* = 3.16, *df* = 9, *p* = 0.012) for brainstem segmentation.

## Discussion

Brain structure segmentation is an important neuroimaging method and various methods and tools have been proposed for reliable and accurate segmentation of brain areas [24,52,53]. SegAdapter is a unique tool that does not perform segmentation by itself, as do most of the other methods, but rather it corrects consistent errors that frequently occur in automated segmentation. In the current study, we evaluated the tool’s utility in correcting segmentation errors from FreeSurfer as well as discrepancies in the boundary definitions for the cerebellum and brainstem. We discovered the excellent performance of the tool for correcting the erroneous inclusion of dura and surrounding tissues in cerebellar labeling and for extending the brainstem boundary to include the substantia nigra. Both corrections required the evaluation of much more extensive areas (dilation radius of 6 voxels) contrasting to its previous applications typically involving only a few voxels around the segmentation of the hippocampus, brain, or brain tissues (i.e. the gray matter and the white matter) [31,32]. Using only 5 scans in the training set, the Dice coefficient against manual segmentation ranged 0.976–0.982 for the cerebellum and 0.951–0.957 for the brainstem in our four groups of participants (younger and older healthy participants and older participants with brain atrophy because of aging or neurodegenerative process). The Dice coefficient improved 0.019–0.024 over FreeSurfer segmentation for the cerebellum and 0.124–0.142 for the brainstem. Using 2 scans in the training set (with increased sampling rate) resulted in minor reductions in Dice coefficient, which were ≤0.002 for the cerebellum and ≤0.005 for the brainstem. The bias in the volumes generated by FreeSurfer also reduced substantially from 5% to 1% higher for the cerebellum and 25% to 3% lower for the brainstem.

Our results demonstrate the utility of SegAdapter in correcting segmentation errors from FreeSurfer in the cerebellum and brainstem and indicate its potential usage in other segmentation tasks. SegAdapter has been applied to improve segmentations of the hippocampus and amygdala, as well as brain extraction, brain tissue segmentation and whole brain segmentation [31,32,54–57]. FreeSurfer, a popular neuroimaging tool, is commonly used for surface-based cortical reconstruction and subcortical segmentation; however, its performance and anatomical definitions may not be ideal [31,58–60]. The corrective learning method can be easily appended to the existing FreeSurfer pipeline (or other tools) by performing manual editing of FreeSurfer segmentation in a small portion of the scans, followed by applying changes to the remaining scans through corrective learning. Residual errors can be further corrected manually if necessary, which requires much less training and effort compared to manual segmentation from scratch or from FreeSurfer segmentation.

The combination of applying automated segmentation and corrective learning is particularly beneficial for large scale neuroimaging studies where a large quantity of scans is acquired across different populations. Our study showed the robustness of the method in scans acquired using different head coils and in participants with brain atrophy because of aging or neurodegenerative process. Using scans acquired with different head coils or from different populations for the training set only reduced Dice coefficient < 0.01 for the two structures compared to when the training set contained the scans acquired with the same head coil and from the same population. Although the older control group showed statistically significant reductions in Dice coefficient, the amount of reduction (< 0.01 in Dice coefficient) was trivial.

Our method of applying corrective learning to FreeSurfer segmentations of the cerebellum and brainstem produced results (Dice coefficient 0.976–0.982 for the cerebellum and 0.951–0.957 for the brainstem) that were among the best of the reported Dice coefficients for automated segmentation against manual segmentation. The prior reported Dice coefficients in the literature have ranged from 0.850 to 0.983 for the cerebellum and 0.830 to 0.952 for the brainstem [25,26,28,30,32,39,53,61–64]. The best performance reported so far (i.e. 0.983 for the cerebellum and 0.952 for the brainstem) came from atlas-based Atropos [26], which employed expectation maximization algorithm to solve Bayesian modelling of brain segmentation problems. However, both our manual segmentation, which served as the ground truth, and the automatic correction were based on Freesurfer’s output. Many of the cerebellar and brainstem labels were not edited, which would produce 100% overlap with automatic correction. Thus, using Dice coefficient to measure the performance of our method may bias towards better performance compared to methods where manual segmentation was generated completely independently from automatic segmentation. Future studies should investigate whether applying corrective learning on other automated tools further improves performance. In addition, Avants el al., 2011 reported the benefit of using N4ITK [65] to correct MRI intensity variation before segmentation. Although FreeSurfer has its own procedure to correct bias field, our unpublished data showed superior brain tissue segmentation in FSL using N4ITK corrected versus FreeSurfer corrected scans. Thus supplying N4ITK-corrected scans to FreeSurfer pipeline may provide additional benefits. More work can also be conducted to evaluate the utility of corrective learning on segmenting the cerebellum in lobules as well as the brainstem in major subdivisions available in the future releases of FreeSurfer [52]. Freesurfer generated and manually corrected cerebellum and brainstem masks, along with the corresponding MPRAGE scans of 20 healthy controls are freely available at https://github.com/jyiwang/segMethod2016.

## Conclusions

We demonstrated significant improvement in segmentation accuracy relative to manual correction by applying machine-based corrective learning to automatically generated segmentations of the cerebellum and brainstem. The combination of automated segmentation and corrective learning produced one of the best results amongst the published data. Our study extended the utility of corrective learning from fixing segmentation errors typically only a few voxels around the boundary to large segmentation errors up to 6 voxels away from the boundary. The method is robust against differences in head coil use during image acquisition and to brain atrophy due to aging or neurodegenerative process, and could be widely applicable to improvement of accuracy and amendment of disagreement in segmentation protocols in brain structures other than the cerebellum and brainstem.
